# Vertical segmental anterior mandibular distraction to aid closure of a severe anterior open bite associated with an accentuated reverse curve of Spee

**DOI:** 10.1177/14653125211000056

**Published:** 2021-03-24

**Authors:** Peter Fowler, Jennifer Haworth, Leon Steenberg

**Affiliations:** 1 Hospital Dental Department, Christchurch Hospital, Christchurch, New Zealand; 2 Department of Child Dental Health, Bristol Dental School, University of Bristol, Bristol, UK; 3 St George’s Hospital, Christchurch, New Zealand

**Keywords:** vertical segmental anterior mandibular distraction, anterior open bite, reverse curve of Spee

## Abstract

**Background:**

The correction of severe anterior open bite is technically challenging, often requiring the use of complex orthodontic mechanics and/or orthognathic surgery and has a relatively high risk of relapse. A marked reverse curve of Spee in the lower arch presents additional challenges when correcting a severe anterior open bite.

**Methods and Materials:**

A 22.2-year-old Caucasian man presented with concerns relating to poor anterior occlusion associated with a 1.3-cm anterior open bite. There was an accentuated reverse curve of Spee to the lower arch, an increased maxillary-mandibular plane angle and increased lower face height. Multidisciplinary treatment involving the use of segmental anterior mandibular distraction to level the curve of Spee before undertaking a Le Fort I posterior maxillary impaction is described in this case report.

**Results:**

Long-term post-treatment records showed stable anterior open bite correction.

**Conclusions:**

This case report illustrates the successful use of segmental anterior mandibular vertical distraction followed by conventional Le Fort I posterior impaction surgery to correct a severe anterior open bite associated with an accentuated reverse curve of Spee and high maxillary-mandibular plane angle.

## Introduction

Successful management of anterior open bite (AOB) is one of the greatest challenges in orthodontics. An AOB can vary in severity and occurs when the occlusal planes of maxillary and mandibular dentitions fail to overlap anteriorly (Kim, 1987). The aetiology of AOB can be multifactorial, with digit or cloth sucking, lip or tongue habits, accentuated reverse curve of Spee, airway obstruction and skeletal growth abnormalities all implicated either singularly or in combination. Correct identification of the aetiology is thought to improve the chance of successful treatment (Ngan and Fields, 1997).

Open bites are dental or skeletal in origin. Skeletal open bites refer to the hyperdivergent growth pattern observed in those with a backward and downward growth rotation (Proffit et al., 2000). These patients tend to exhibit underdevelopment of masseter and temporalis muscles and their teeth are subject to mesial component of forces, leading to bidental protrusion (Sassouni, 1969). The palatal plane slopes upwards and forwards (Kim, 1987). The axial inclination of the teeth in relation to the occlusal plane is implicated in the curved occlusal plane. The direction of the eruption path of teeth is likely to be multifactorial and although genetics play a part, environmental factors which may have an influence include strong anterior component of forces and crowded dentitions. These may account for the marked mesial inclination of teeth noted in open bite cases (Kim, 1987).

The development of the curve of Spee is usually completed once the second permanent molars erupt and remains relatively stable into adulthood (Marshall, 2008). Craniofacial morphology has a minor influence on its form (Farella et al., 2002). In the mandibular arch, a reverse curve of Spee is associated with untreated open bite cases (Trouten et al., 1983). Inadequate compensation accounts for the position of the lower incisors at or below the functional occlusal plane, resulting in the reverse curve of Spee (Trouten et al., 1983). A marked reverse curve of Spee in the mandibular arch, alongside a symmetrical AOB and bimaxillary incisor inclination, may be indicative of endogenous tongue thrust or macroglossia (Naini and Gill, 2017). Generally, however, a tongue thrust swallow is thought to be an adaptation to the space between the teeth in an AOB, not the cause (Proffit et al., 2000).

Management of open bite in growing patients is aimed at minimising eruption of posterior teeth, or actively intruding molar teeth, using techniques such as high pull headgear or vertical pull chin cup treatment (Proffit et al., 2000). There is minimal available evidence regarding the management of a mandibular reverse curve of Spee in open bite treatment. In a report by Trouten et al. (1983), open bite patients who were successfully treated were found to have a positive occlusal curve at the end of treatment, with a slight post-retention ‘rebound’.

Skeletal open bites in adult patients have been treated successfully using multiloop edgewise appliances with anterior vertical elastics (Kim et al., 2000), lower molar intrusion using skeletal anchorage systems (Umemori et al., 1999), posteriorly placed repelling magnets (Meral and Yüksel, 2003) or using clear aligners (Guarneri et al., 2013). Joint orthodontic-surgical procedures are required for more severe skeletal open bite in non-growing patients (Ngan and Fields, 1997). Surgical management may include differential posterior impaction of the Le Fort I osteotomised maxilla, segmental impaction of the posterior maxilla or isolated mandibular surgery (Naini and Gill 2017). Silberman et al. (1972) described the use of bimaxillary anterior vertical osteotomies and partial glossectomy for a patient presenting with a skeletal AOB associated with bimaxillary protrusion and macroglossia. For those patients with vertical maxillary excess, superior repositioning of the maxilla, by total or segmental maxillary osteotomies, may be required. The most stable ranked orthognathic procedure in the treatment of skeletal AOB includes superior repositioning of the maxilla allowing the mandible to ‘auto’ rotate upward and forward for those patients with increased lower face heights (Proffit et al., 2000).

Distraction osteogenesis has been defined as ‘a method of biologically creating new bone between surgically cut bone surfaces that are separated in a controlled manner by incremental traction’ (Barber et al., 2018). It is a versatile technique that increases the length of bones and can theoretically be applied in all three planes of space, changing the form of bones (Ilizarov, 1988). Greater movements are possible than with conventional orthognathic surgery, although this can be at the expense of loss of precision of movement (Proffit et al., 2000). The indications for distraction osteogenesis most commonly include congenital anomalies, trauma or pathology, including mandibular/maxillary distraction for mandibular/maxillary hypoplasia/retrognathia (Cope et al., 1999). Although mandibular segmental vertical distraction osteogenesis has been used to reconstruct vertically deficient alveolar ridges before implant placement (Saulacić et al., 2007), it has not been regularly used as an aid in the treatment of severe AOB.

The aim of the present report is to describe the multidisciplinary treatment of a patient who presented with a severe AOB with an accentuated mandibular reverse curve of Spee, an increased maxillary-mandibular plane angle and increased lower face height. Treatment involved orthodontic therapy and the use of segmental anterior mandibular vertical distraction to level the curve of Spee before undertaking a Le Fort I maxillary posterior impaction. The patient was monitored over 8.2 years. Written informed consent was obtained from the patient for the publication of the clinical records.

## Case history

A 22.2-year-old Caucasian man presented with concerns relating to poor anterior occlusion and difficulty eating. The anterior occlusal relationship had been present since adolescence. There was no history of temporomandibular joint dysfunction, facial trauma or obstructive sleep apnoea, and the patient had no significant medical history.

## Assessment

### Clinical examination

**Figure 1.**
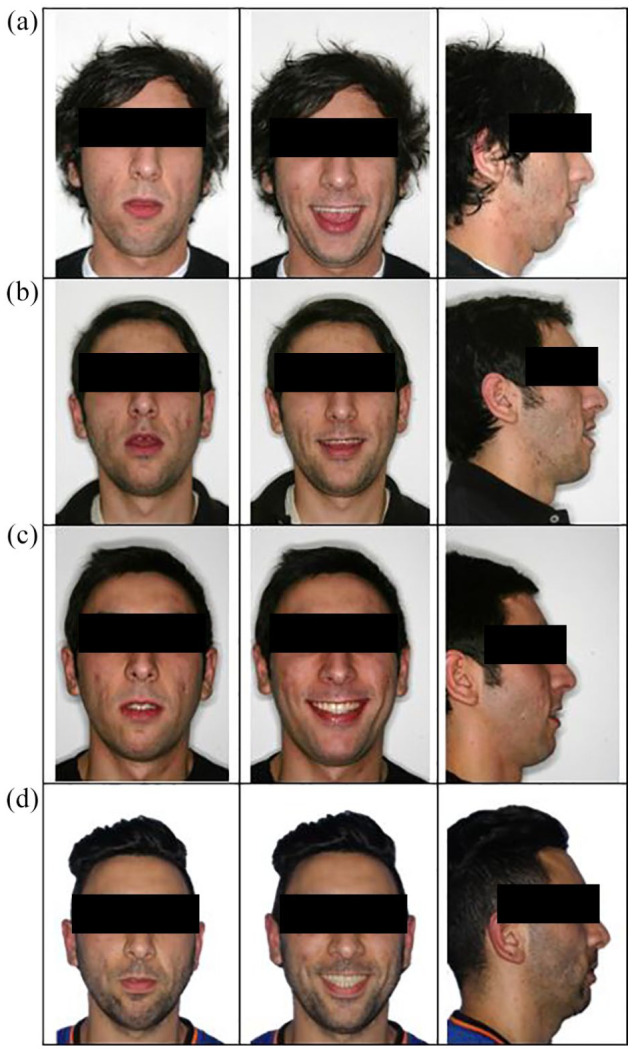
Extra-oral images. (a) Pre-treatment. (b) Distraction consolidation. (c) Brace removal. (d) 8.2-year follow-up.

**Figure 2.**
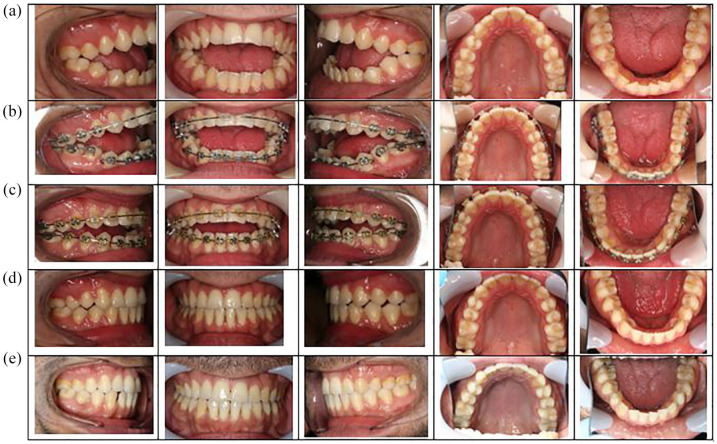
Intra-oral photographs. (a) Pre-treatment. (b) Early distraction. (c) Post distraction / pre-Le Fort 1. (d) Brace removal. (e) 8.2-year follow-up.

The patient had a Class 2 skeletal relationship with retrognathic mandible, an increased Frankfort-mandibular plane angle (FMA) and an excessive lower face height. There was facial symmetry, and his lips were incompetent with 3-mm maxillary tooth display upon smiling (Figure 1a). There was very mild upper anterior irregularity and mild lower anterior crowding with the lower third molars partially erupted but potentially impacted. In occlusion, there was a 5-mm overjet and a 1.3-cm AOB that extended posteriorly to the first molars. There was an accentuated reverse curve of Spee in the lower arch resulting in essentially a dual occlusal level where the anterior teeth were stepped down from the posterior teeth distal to the first premolars. The molar relationship was ¼ Class III and there was a 2-mm midline shift to the left in the lower arch (Figure 2a).

### Radiographic examination

**Figure 3.**
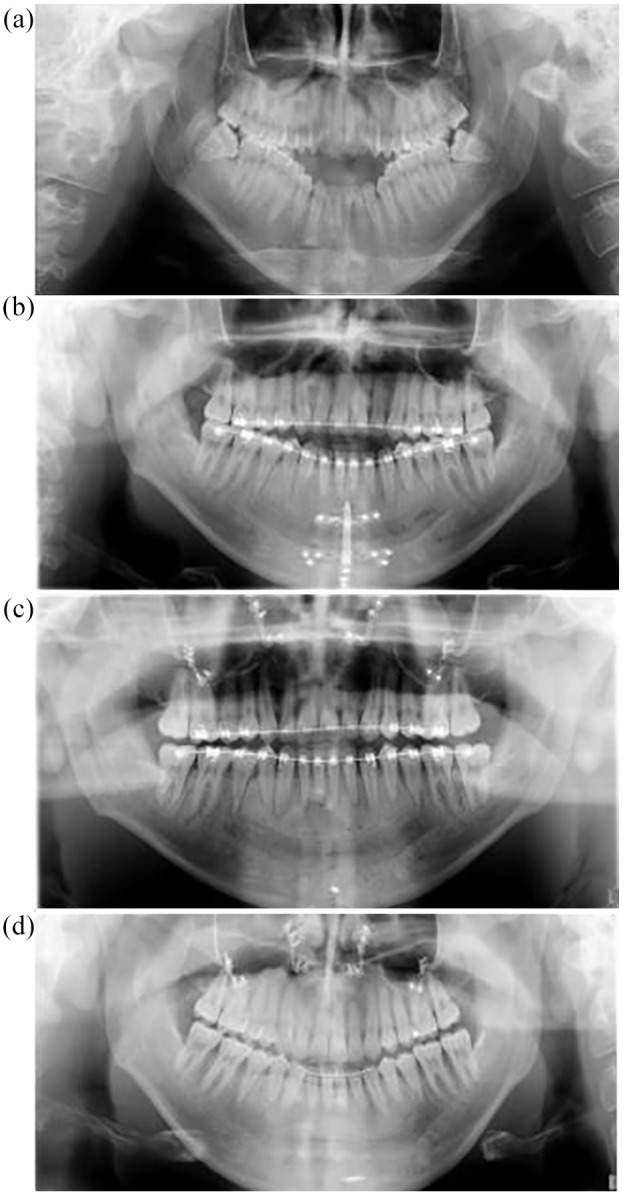
DPT images. (a) Pre-treatment. (b) Post distraction. (c) Post Le Fort 1. (d) Brace removal.

**Figure 4.**
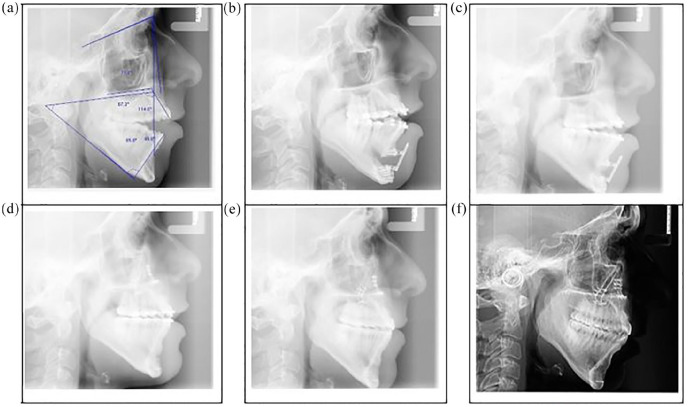
Lateral cephalograms. (a) Pre-treatment. (b) Active distraction. (c) Distraction consolidation. (d) Immediate post LeFort 1 impaction. (e) Brace removal. (f) 8.2-year follow-up.

The pre-treatment DPT confirmed normal condyle anatomy and potential impaction of the lower third molars (Figure 3a). The lateral cephalometric radiograph (Figure 4a) revealed a mild Class 2 skeletal relationship, an excessive maxillary-mandibular plane angle and increased lower face height with a dual mandibular occlusal table associated with the reverse curve of Spee (Table 1).

**Table 1.** Lateral cephalogram analysis: (A) pre-treatment; (B) pre-Le Fort I; (C) brace removal; (D) 8.2-year follow-up.

Skeletal AP	A	B	C	D
SNA (º)	71.7	72.4	75.3	74.4
SNB (º)	67.3	69.6	71.7	71.8
ANB (º)	4.4	2.8	3.6	2.6
*Dental*				
U1 - Palatal plane (º)	114.0	118.6	119.0	117.0
L1 - GoGn (º)	85.8	81.5	75.4	76.8
Interincisal Angle (U1-L1) (º)	116.9	128.3	127.0	127.7
Overbite (mm)	−13.1	−4.0	0.4	0.5
Overjet (mm)	5.0	1.7	3.0	1.9
*Facial proportions*				
Post/Ant face height (%)	56.2	56.6	54.1	56.0
AUFH (mm)	64.8	64.5	62.5	63.9
ALFH (mm)	74.3	74.9	76.7	73.6
*Vertical*				
Maxillary-Mandibular angle (PP-MP) (º)	46.8	45.5	39.0	37.4
Y-axis (SGn - SN -7) (º)	79.2	77.2	76.2	76.4
MP - SN (º)	60.0	59.4	58.9	58.1
*Facial convexity*				
Convexity (NA-APo) (º)	11.5	16.5	11.6	10.4
Holdaway angle (NB to H-line) (º)	19.3	12.7	11.5	14.8

ALFH, anterior lower facial height; AUFH, anterior upper facial height.

### Treatment plan and progress

Due to the excessive nature of the AOB, a combined orthodontic and orthognathic surgical treatment approach was planned with the patient. However, the correction of the dual occlusal level associated with the accentuated reverse curve of Spee in the lower arch before undertaking conventional Le Fort I maxillary posterior impaction and small advancement presented treatment planning challenges. Orthodontic extrusion of the lower labial segment was considered to be potentially unstable and over the longer term it was likely to result in the return of the AOB if there was relapse to the extrusive incisor movements. Intrusion of the lower posterior teeth was considered technically challenging even with the use of temporary anchorage devices (TADs). The option of using a vertical mandibular distractor in the anterior segment to level the curve of Spee was discussed with the patient. Although this proposed treatment option would level the curve of Spee and avoid the need for a bone graft, there would be a further increase to the vertical height of the anterior mandible, which in turn may necessitate reduction genioplasty surgery.

Upper and lower fixed appliances (0.022-inch preadjusted edgewise) were placed to level and align the upper arch, maintaining similar incisor inclinations while lower alignment and levelling was undertaken with sectional mechanics to maintain two occlusal planes (Figure 2b). A vertical distractor (Martin Track 1 Plus) was placed with the aim of elevating the anterior mandibular segment mesial to the lower second premolars to achieve one lower occlusal plane. The partially erupted, mesially impacted lower and opposing upper third molars were removed at the time of distractor placement. Although removal of asymptomatic pathology-free wisdom teeth is not normally recommended in the UK (NICE, 2000), other countries follow different protocols and the patient and surgeon opted jointly for removal of these teeth in this case. The activation of the distractor was initiated 48 h after the distractor placement surgery to a prescribed elevation of 1.0 mm per day by twice daily turns of the distractor arm. Approximately 1 cm of elevation was achieved during the activation and consolidation distraction phase, which was 8.5 months in duration (Figure 3b). Minimal discomfort relating to the distractor was reported by the patient and there was only mild temporary disturbance to sensation of the lower lip. At the completion of the distraction phase, a reduced residual open bite and a slight overjet remained (Figures 2c and 4c).

Using continuous coordinated 0.019 × 0.025-inch stainless-steel arch wires, arch coordination was achieved before undertaking a conventional Le Fort I posterior impaction of 4 mm and advancement of 4 mm to establish a positive overjet and overbite. The distractor was removed at the time of the Le Fort 1 operation, although one screw was inadvertently left in situ (Figure 3c). Final detailing of the occlusion was required using light vertical elastics (250 g force) before removal of appliances four months postoperatively. Vacuum formed retainers and a lower 3-3 bonded retainer were provided (Figures 1c, 2d, 3d and 4e). Long-term retention supervision was provided with follow-up records collected 8.2 years after appliance removal (Figures 1d, 2e and 4f).

### Treatment outcome

A clinical examination at appliance removal and at subsequent retention visits, including the 8.2-year follow-up appointment, showed satisfactory facial and occlusal relationships with successful closure of the excessive AOB (Figures 1d, 2e and 4f). Plans to carry out an additional vertical reduction genioplasty to reduce the mandibular anterior vertical dimension and improve lip competency were declined by the patient.

Pre- and post-treatment lateral cephalogram measurements showed slight increases in SNA and SNB with a small reduction in ANB differences. The vertical proportions were similar with a small reduction in maxillary-mandibular plane angle (Table 1 and Figure 4). Longer-term clinical and lateral cephalometric findings show slight lower anterior proclination/malignment with stable positive overjet and stable, but reduced overbite (Figures 2e and 4e). Minor root resorption to the mandibular premolars was noted on the progress and end of treatment DPT (Figure 3c and d).

## Discussion

This patient presented with challenging clinical features, including an excessive AOB associated with an accentuated reverse curve of Spee to the lower arch and an increased maxillary-mandibular plane angle and increased lower face height. Surgical correction with the use of a segmental anterior mandibular vertical distractor was considered a stable option to level the lower occlusal plane before undertaking conventional Le Fort I posterior impaction and small advancement. At the completion of treatment, a dramatic improvement occlusal outcome was achieved.

Over 1 cm of vertical distraction was achieved in the anterior mandibular segment with good bone infill occurring during the consolidation phase, avoiding the requirement for bone grafting that would have been required if conventional surgical elevation was undertaken. Other treatment considerations could have included a concurrent conventional lower labial segment osteotomy and vertical reduction genioplasty using the bone to fill the osteotomy bony defect, as well as the placement of TADs in the maxilla for skeletal anchorage to intrude the posterior segment (Cousley, 2014). This may have reduced or negated the use of conventional Le Fort 1 impaction. Although most Class 2 skeletal AOB can be successfully treated with conventional maxillary Le Fort 1 and mandibular bilateral sagittal split osteotomy, due to the severity of the presenting AOB and accentuated reverse curve of Spee, a novel approach was undertaken to achieve a satisfactory and stable occlusal result.

Longer-term post-treatment records show stable open bite closure was achieved with the elevation of the mandibular anterior segment as well as maxillary posterior impaction. The vertical facial proportions remain similar to pre-treatment values with lip competency achieved only through mentalis activity. This could be improved with the use of a vertical reduction genioplasty carried out at the time of the Le Fort 1 but this has been declined by the patient.

## Summary

The present case report demonstrated the multidisciplinary treatment of a severe AOB in which the use of both vertical segmental anterior mandibular distraction and a conventional Le Fort I osteotomy were required. The vertical distraction was a novel approach to correct a dual occlusal plane resulting from an accentuated reverse curve of Spee. The establishment of a positive overbite and overjet was stable over a 8.2-year period.
